# Experimental Autoimmune Neuritis Nerve Demyelination Is Attenuated by Blocking JAK2/STAT3 Signaling Pathway in Rats

**DOI:** 10.1002/brb3.70566

**Published:** 2025-05-18

**Authors:** Rumeng Zhou, Shuping Liu, Yue Liu, Yin Liu, Rong Fu, Jiajia Yao, Zuneng Lu

**Affiliations:** ^1^ Department of Neurology Renmin Hospital of Wuhan University Wuhan Hubei China

**Keywords:** AG490, experimental autoimmune neuritis, JAK‐STAT signaling pathway, treatment

## Abstract

**Background:**

Guillain‒Barré syndrome (GBS) is an immune‐mediated peripheral neuropathy in which inflammatory cells and cytokines participate. The JAK‐STAT signaling pathway is a major pathway involved in cytokine signal transduction, but the role of this pathway in GBS is not clear. AG490 is a tyrosine kinase inhibitor that specifically inhibits JAK2 activity and downregulates STAT3 phosphorylation. The aim of this study was to investigate the function of the JAK2/STAT3 pathway in a rat model of experimental autoimmune neuritis (EAN).

**Methods:**

Lewis rats were divided into three groups: the control, the EAN, and the AG490 groups. The EAN and AG490 groups were immunized with P2 peptide to create the EAN models, while the control group received an equal volume of vehicle solution without P2 peptide. Starting from Day 5 post‐immunization (PI), the AG490 group was administered AG490 (10 mg/kg) every other day, while the control and EAN groups received an equal volume of vehicle solution without AG490. All rats were weighed and evaluated according to the EAN function score (1–10) by two investigators. Rats were sacrificed on Day 16 PI, and the sciatic nerves were examined by light microscopy, indirect immunohistochemistry, and western blotting.

**Results:**

AG490‐treated rats had improved clinical scores compared with those of EAN rats. Hematoxylin and eosin (H&E) and CD45 staining showed significant inflammatory infiltration of the sciatic nerve in the EAN group compared with the control group, and demonstrated reduced inflammatory infiltration in the AG490 group. Luxol fast blue (LFB) staining showed a reduction of myelin loss in the AG490 group compared with the EAN group. The levels of TGF‐β1, IFN‐γ, and IL‐6 increased in the EAN group and showed a significant decrease in rats treated with AG490. The JAK2‐STAT3 signaling pathway was activated in EAN rats, and the AG490 group showed decreased expression levels of JAK2, p‐JAK2, and p‐STAT3 compared with those of the EAN group. Immunofluorescence also showed a decrease in the levels of p‐JAK2 and p‐STAT3 in the sciatic nerve of EAN rats.

**Conclusions:**

The JAK2/STAT3 signaling pathway is involved in the pathogenesis of EAN, and inhibition of this pathway can reduce the inflammatory response in EAN rats. Despite the limitations in extrapolating EAN findings to human GBS, this study provided new insights into the pathogenesis and potential therapeutic targets of human GBS.

## Graphical abstract

We developed the EAN model in Lewis rat, and investigated the effect of the JAK2/STAT3 pathway inhibitor AG490 on EAN. AG490 reduced the inflammatory infiltration and mitigated demyelination damage in EAN rats through the inhibition of JAK2/STAT3 signaling pathway.

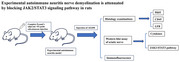

## 1 Introduction

Guillain‒Barré syndrome (GBS) is an immune‐mediated peripheral neuropathy, characterized by acute paralysis of the limbs and the absence of tendon reflexes. GBS is the leading cause of acute flaccid paralysis worldwide, with a higher incidence in males than in females (Shahrizaila et al. 2021; van Doorn et al. 2008; Willison et al. 2016). The pathogenic mechanism of GBS is not yet clear, and both humoral and cellular immunity contribute to the occurrence of GBS (van Doorn et al. 2008). Demyelinating and axonal damage are the main pathologies of GBS, resulting in two different subtypes (Shahrizaila et al. 2021; Willison et al. 2016). Although immunotherapies have been used to treat GBS, the prognosis of the disease has not improved substantially.

Experimental autoimmune neuritis (EAN) is a well‐established animal model of GBS, as it exhibits clinical and pathological features closely resembling those observed in human GBS (Brostoff et al. 1977; Waksman and Adams 1955; Saida et al. 1979). The rat model of EAN, which is induced by immunizing Lewis rats with P2 peptide, is widely used to elucidate the pathogenesis of the demyelinating subtype of GBS (Brostoff et al. 1977). However, the pathogenic mechanisms and treatments identified in the rat model of EAN do not always apply to human GBS (Asbury and McKhann 1997). The EAN rat model is induced by exogenous P2 peptide, which is not the case for GBS patients, and this model only represents the demyelinating subtype of GBS.

The immune response in both EAN and demyelinating GBS is primarily mediated by T cells, and CD4+ T cell subsets play the key role in the immunoregulation of GBS and EAN (Zhang et al. 2013). Imbalances in CD4+ T cell responses have been reported in GBS patients (Shi et al. 2018;Súkeníková et al. 2024). Modulating the differentiation of CD4+ T cells could alleviate EAN (Xie et al. 2024; Liu et al. 2018). All these findings suggest that regulating the differentiation of CD4+ T cells may be a potential therapeutic target for EAN and GBS. The JAK/STAT signaling pathway is a widely expressed intracellular signaling mechanism that plays a key role in CD4+ T cell differentiation (Villarino et al. 2017). Cytokines, such as IL‐6, have been shown to activate the JAK/STAT signaling pathway, with STAT3 potentially driving the differentiation of naive CD4+ T cells into Th17 cells (Wu et al. 2017). STAT3 gene expression was significantly increased in the lymphocytes of GBS patients (Debnath et al. 2018), and the level of phosphorylated STAT3 was elevated in EAN models (Shao et al. 2023). These findings suggest that the JAK/STAT signaling pathway may be involved in the production of EAN and GBS. In addition, the JAK‐STAT signaling pathway participates in multiple neurological diseases, and inhibition of the JAK‐STAT pathway can help mitigate disease severity (Yan et al. 2018; Kooshki et al. 2023; Egwuagu and Larkin 2013).

AG490 is a specific inhibitor of the JAK‐STAT pathway and can block the phosphorylation of JAK2 and STAT3. Several studies have shown that AG490 can reduce inflammation and apoptosis (Banerjee et al. 2017; Li et al. 2019; Xiao et al. 2020). Therefore, we hypothesize that the JAK‐STAT signaling pathway participates in the occurrence of EAN and that AG490 could produce a therapeutic effect on EAN by inhibiting the JAK‐STAT pathway.

## 2 Materials and Methods

### 2.1 Animals and EAN Model

Thirty male Lewis rats (5–7 weeks old, body weight of 160–180 g) were purchased from Beijing Vital River Laboratory Animal Technology Co., Ltd. The sample size was determined based on the variance observed in pilot studies and previous experiments. Rats were raised in a specific pathogen‐free (SPF) laboratory for one week and fed standard water and rodent chow throughout the experiments. The rats were randomly divided into three groups using a random number table: The control group (*n* = 10), the EAN group (*n* = 10), and the AG490 group (*n* = 10). The Animal Ethics Committee of Renmin Hospital of Wuhan University reviewed and approved the experiments.

To induce the EAN model, rats in the EAN and AG490 groups were immunized by subcutaneous injection at the base of the tail with 200 µL of inoculum containing 400 µg of P2_57–81_ peptide (Phe‐Lys‐Asn‐Thr‐Glu‐Ile‐Ser‐Phe‐Lys‐Leu‐Gly‐Gln‐Glu‐Phe‐Glu‐Glu‐Thr‐Thr‐Ala‐Asp‐Asn‐Arg‐Lys‐Thr‐Lys, GL Biochem Ltd. China) and 2 mg of *Mycobacterium tuberculosis* H37RA (Difco, USA) in 100 µL of incomplete Freund's adjuvant (Sigma, USA) and 100 µL of physiological saline. Meanwhile, the rats in the control group were injected with 200 µL of inoculum containing 2 mg of Mycobacterium tuberculosis H37RA, 100 µL of incomplete Freund's adjuvant, and 100 µL of physiological saline.

The rats were weighed and the clinical scores were assessed by two independent observers in a blinded manner using the following scale before immunization (Day 0) and every day of the experiment: 0 = normal; 1 = reduced tonus of the tail; 2 = limp tail; 3 = absent righting reflex; 4 = gait ataxia; 5 = mild paresis of the hind limbs; 6 = moderate paraparesis; 7 = severe paraparesis or paraplegia of the hind limbs; 8 = tetraparesis; 9 = moribund; and 10 = death.

### 2.2 Treatment With AG490

AG490 (MCE, China) was dissolved in a 2% DMSO solution diluted in physiological saline to prepare the working solution. The rats in the AG490 group were treated with AG490 (10 mg/kg) by intraperitoneal injection (i.p.) every other day from Day 5 PI to Day 15 PI, while the rats in the control and EAN groups were treated with an equal volume of 2% DMSO in physiological saline.

### 2.3 Histological Analysis

Sciatic nerves from all groups were collected and fixed in 4% paraformaldehyde in phosphate‐buffered saline (PBS) on Day 16 PI, then embedded in paraffin, sectioned into 4 µm slices, and routinely stained with H&E and LFB to evaluate inflammation and demyelination. Starting from the center of each specimen, six to nine sections spaced 1 mm apart were captured using a fluorescence microscope (Olympus, Tokyo, Japan) at 200× magnification (per field of view, 400 × 400 µm^2^). The images were measured independently by two observers in a blinded manner using ImageJ software. Inflammatory cell infiltration was quantified as inflammatory cell counts per field of view. On LFB staining, the myelinated area was stained bright blue (LFB‐positive area), while demyelinated lesions were either stained a lighter blue or remained unstained. The severity of demyelination was quantified as the proportion of LFB‐positive area per field of view.

### 2.4 Immunohistochemistry

Paraffin‐embedded sections were deparaffinized, rehydrated, and heated in sodium citrate antigen retrieval solution (PH 6.0) at 94°C for 10 min. The sections were treated with 3% hydrogen peroxide solution for 10 min and blocked in 5% bovine serum albumin solution for 1 h. For detection of leukocytes, the sections were incubated overnight with rabbit anti‐CD45 (1:300, Servicebio, China) at 4°C. Then, the sections were incubated with HRP‐labeled goat anti‐rabbit IgG (1:200, Servicebio) and developed using 3,3‐diaminobenzidine substrate. The sections were captured and measured using the same methods described in the “Histological analysis” section. Leukocyte infiltration was quantified as CD45‐positive cell counts per field of view.

### 2.5 Immunofluorescence Staining

Paraffin‐embedded sections of the sciatic nerve were also used for immunofluorescence analysis. The tissue sections were incubated with antibodies specific for phospho‐JAK2 (Tyr1007/1008; rabbit polyclonal antibody IgG, 1:100, Wanlei, WL02997) and phospho‐STAT3 (S727; rabbit monoclonal IgG, 1:100, Abclonal, China, AP0715) at 4°C overnight, then washed and incubated with secondary antibodies (Alexa Fluor 488 donkey anti‐rabbit IgG and Alexa Fluor 594 goat anti‐rabbit IgG, 1:200, Antgene, China) for 1 h at room temperature. The sections were captured and measured using the same methods described in the “Histological analysis” section. The expression of p‐JAK2 and p‐STAT3 was quantified as the number of positive cells per field of view.

### 2.6 Western Blot Analysis

Five sciatic nerve samples from each group were lysed in RIPA buffer (Servicebio, China) containing phenylmethylsulfonyl fluoride (PMSF) (Servicebio, China) and a protease inhibitor cocktail (Servicebio, China). The concentrations of the protein lysates were quantified by BCA assay (Beyotime Biotechnology, China). Equal amounts of protein from each group were separated by SDS‒PAGE and then transferred to PVDF membranes. After blocking, the membranes were incubated with primary antibodies against β‐actin (1:5000, Proteintech, China), IL‐6 (1:1000, Affbiotech, China), IFN‐γ (1:100, Santa Cruz, USA), TGF‐β1 (1:200, Santa Cruz, USA), p‐JAK2 (1:1000, Wanlei, China), and p‐STAT3 (1:2000, Abclonal, China) at 4°C overnight. After incubation with primary antibodies, the membranes were washed in TBST 3 times for 5 min per wash and incubated with horseradish peroxidase‐conjugated secondary antibody at a dilution of 1:3000 at room temperature for 1 h. Finally, the bands were visualized with the ChemiDoc XRS+ system (Bio‐Rad, USA), and quantification of the proteins of interest was performed using the ImageJ program.

### 2.7 Statistical Analysis

Statistical analyses were performed with IBM SPSS Statistics v26.0 software. All data are presented as the mean±SD. Continuous variables were examined by Student's *t* tests, categorical variables were examined by ANOVA, and nonparametric tests were used for data that did not conform to a normal distribution. Differences were considered statistically significant at a *p* value < 0.05.

## 3 Results

### 3.1 Clinical Symptoms

**FIGURE 1:**
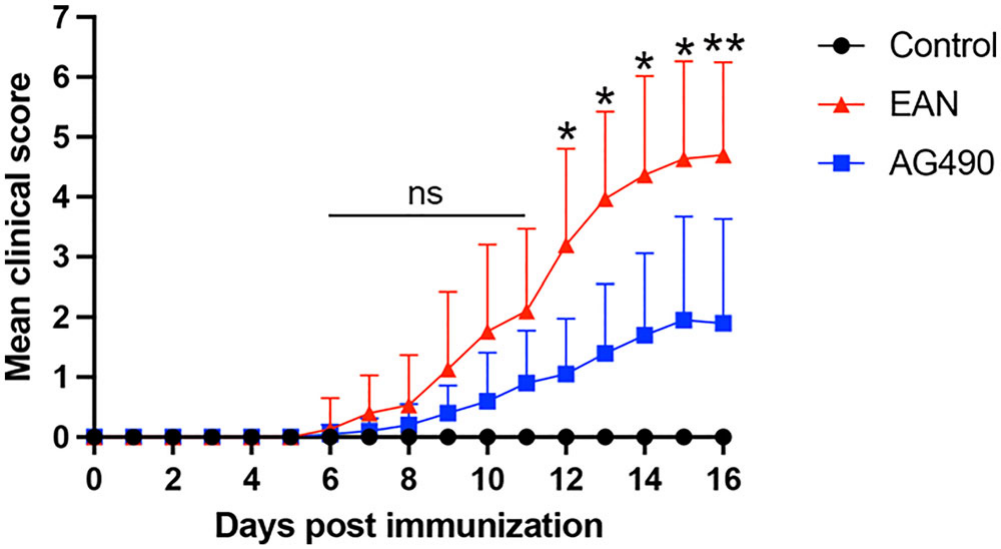
Clinical scores of rats in each group. AG490 was administered every other day from Day 5 to Day 15 post‐immunization (PI). Clinical scores of rats in EAN and AG490 groups increased after immunization. AG490 treatment significantly ameliorated severity in EAN from day 12 PI. Mean ± SD, *n*=10/group. ns, no significant difference, * *p *< 0.05, ** *p *< 0.01, AG490 versus EAN.

All rats that were immunized with the P2 peptide developed clinical symptoms. The median time from immunization to the onset of clinical symptoms was 8.4 ± 1.5 days in EAN rats and 10.2 ± 2.8 days in the AG490 group, and the time from immunization to onset of clinical symptoms was not significantly different between the groups. Moreover, the clinical scores of rats in the AG490 group were significantly lower than those of rats in the EAN group from day 12 PI. The results suggested that AG490 ameliorates severity in EAN (Figure 1).

### 3.2 Effect of AG490 on the Histology of EAN Rats

**FIGURE 2:**
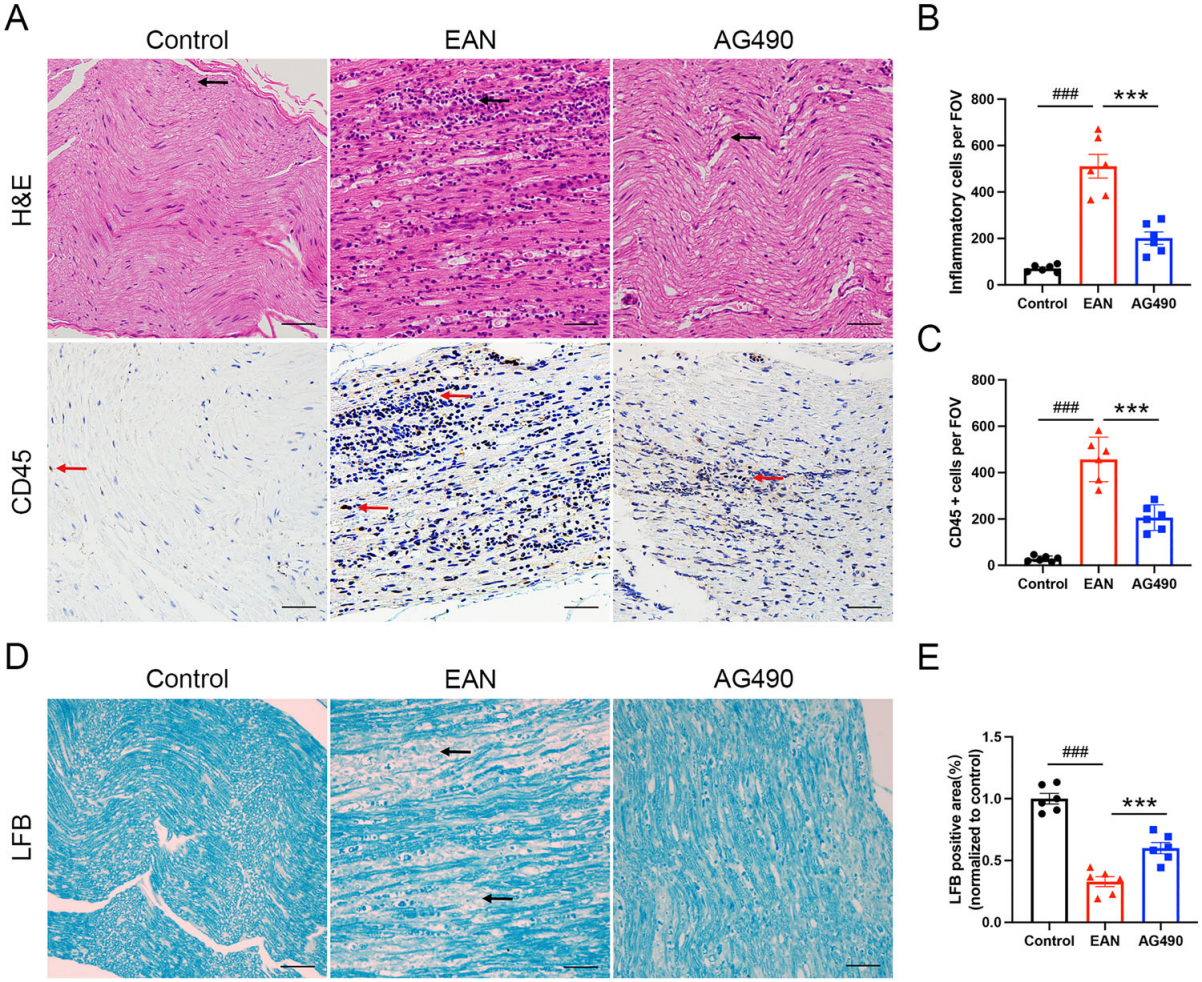
AG490 attenuated the histologic signs in EAN. The sciatic nerves were harvested at day 16 PI in each group for histologic examinations. (A) Representational photomicrographs of H&E staining and CD45 staining in sciatic nerve of each group. Inflammatory cells were indicated by arrows in each group. (B) The quantitative analysis of inflammatory cell infiltration per FOV. (C) The quantitative analysis of CD45‐positive cells per FOV. (D) Representational photomicrographs of LFB staining in the sciatic nerve of each group. Demyelinated lesions in EAN group were indicated by arrows. (E) Relative quantitative analysis of LFB‐positive area ratio per FOV (normalized to control group). Lower LFB‐positive area ratio indicated severe demyelination in sciatic nerves. FOV, field of view. Scale bars: 50 µm. Mean ± SD, *n* = 6/group. **###** *p *< 0.001, Control versus EAN. *** *p *< 0.001, AG490 versus EAN.

To explore the function of AG490 in reducing pathological damage in EAN rats, we used H&E staining, CD45 staining, and LFB staining to evaluate the inflammatory infiltration and demyelination of the sciatic nerve. H&E staining showed substantial inflammatory infiltration in the sciatic nerve of the EAN group, and CD45 staining revealed an increased number of leukocytes in the EAN group. AG490 treatment significantly reduced the number of inflammatory cells in the AG490 group (Fig 2A–C). Compared with control rats, rats in the EAN group exhibited a significantly reduced LFB‐positive area ratio, indicating severe demyelination in the sciatic nerves. AG490 treatment significantly increased the myelinated area in sciatic nerves, as evidenced by a larger proportion of LFB‐positive area (Fig 2D–E). The findings indicated that AG490 alleviated the pathological damage in EAN rats.

### 3.3 AG490 reduced the Level of TGF‐β1、IFN‐γ、IL‐6 in EAN Rats

**FIGURE 3:**
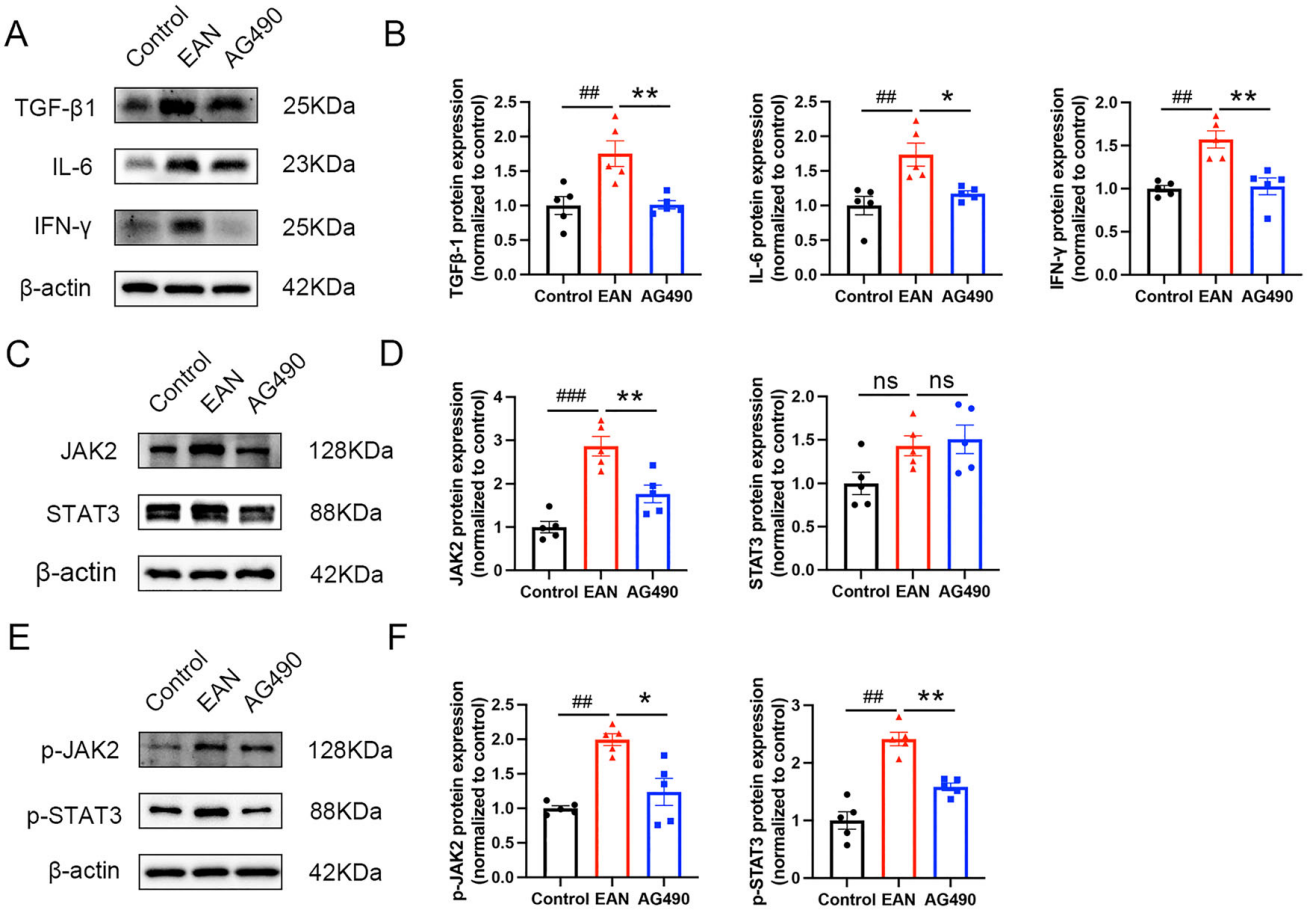
Western blotting showed the protein levels in the sciatic nerves. (A, B) AG490 treatment reduced the levels of TGF‐β1, IFN‐γ, and IL‐6 in EAN rats. (C–F) The levels of JAK2, p‐JAK2, and p‐STAT3 were decreased after treatment with AG490 but the level of STAT3 showed no significant difference. Mean ± SD, *n* = 5/group. **#** *p *< 0.05, **##** *p *< 0.01, **###** *p *< 0.001, Control versus EAN. * *p *< 0.05, ** *p *< 0.01, ns, no significant difference, AG490 versus EAN.

Western blot analysis was used to evaluate the levels of TGF‐β1, IFN‐γ, and IL‐6. The levels of TGF‐β1, IFN‐γ, and IL‐6 were increased in the sciatic nerves in EAN rats; however, the levels of TGF‐β1, IFN‐γ, and IL‐6 were greatly reduced by AG490, indicating that AG490 could alleviate EAN by reducing proinflammatory cytokine levels (Figure 3A–B).

### 3.4 AG490 Alleviated EAN by Inhibiting the JAK2‐STAT3 Signaling Pathway

**FIGURE 4:**
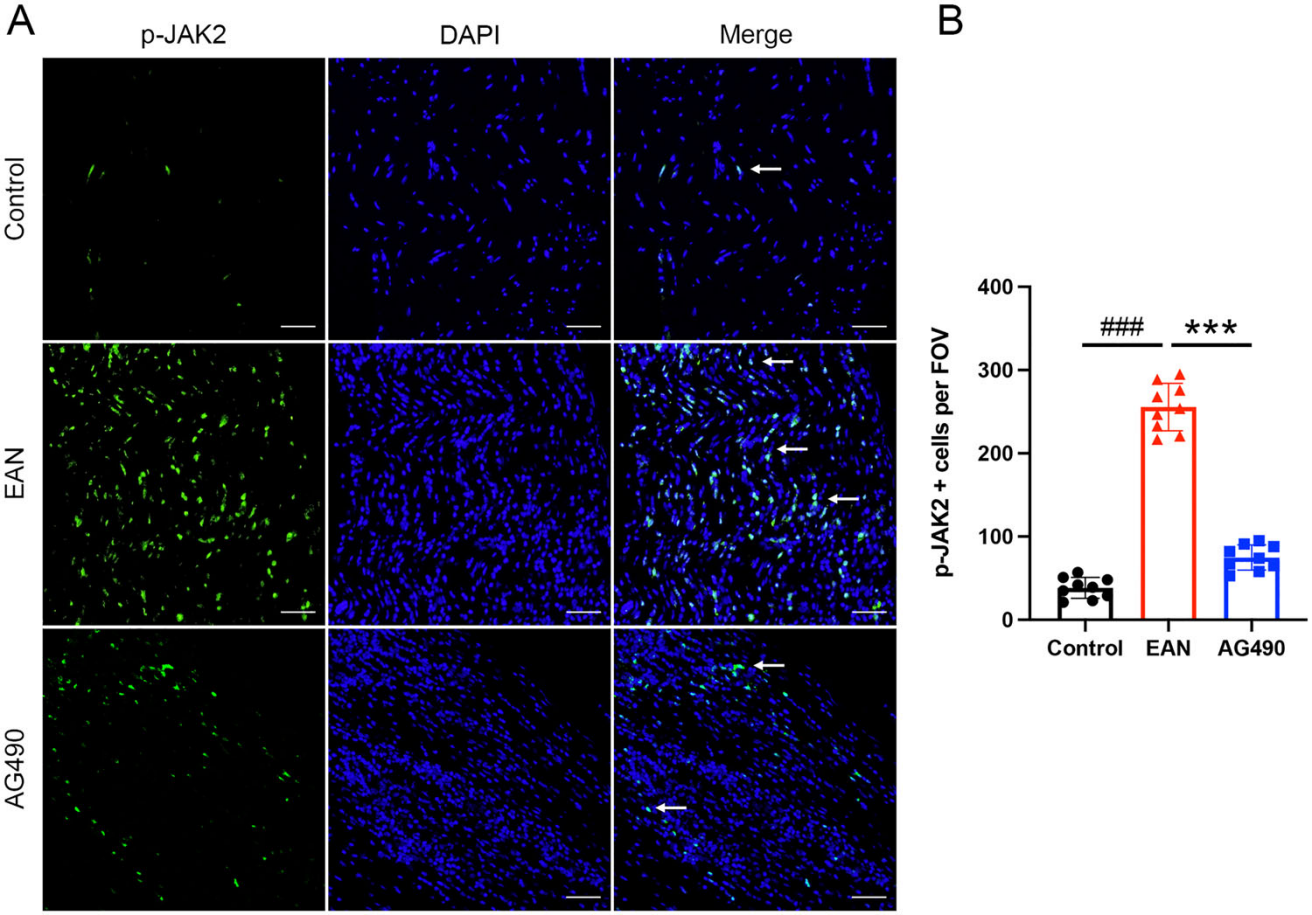
Effect of AG490 on the expression of p‐JAK2 in the sciatic nerves. (A) Representational photomicrographs of p‐JAK2 in the sciatic nerve of each group. p‐JAK2 positive cells were indicated by arrows. (B) The quantitative analysis of p‐JAK2 positive cells per FOV. FOV, field of view. Scale bars: 50 µm. Mean ± SD, *n* = 9/group. **###** *p *< 0.001, Control versus EAN. *** *p *< 0.001, AG490 versus EAN.

**FIGURE 5:**
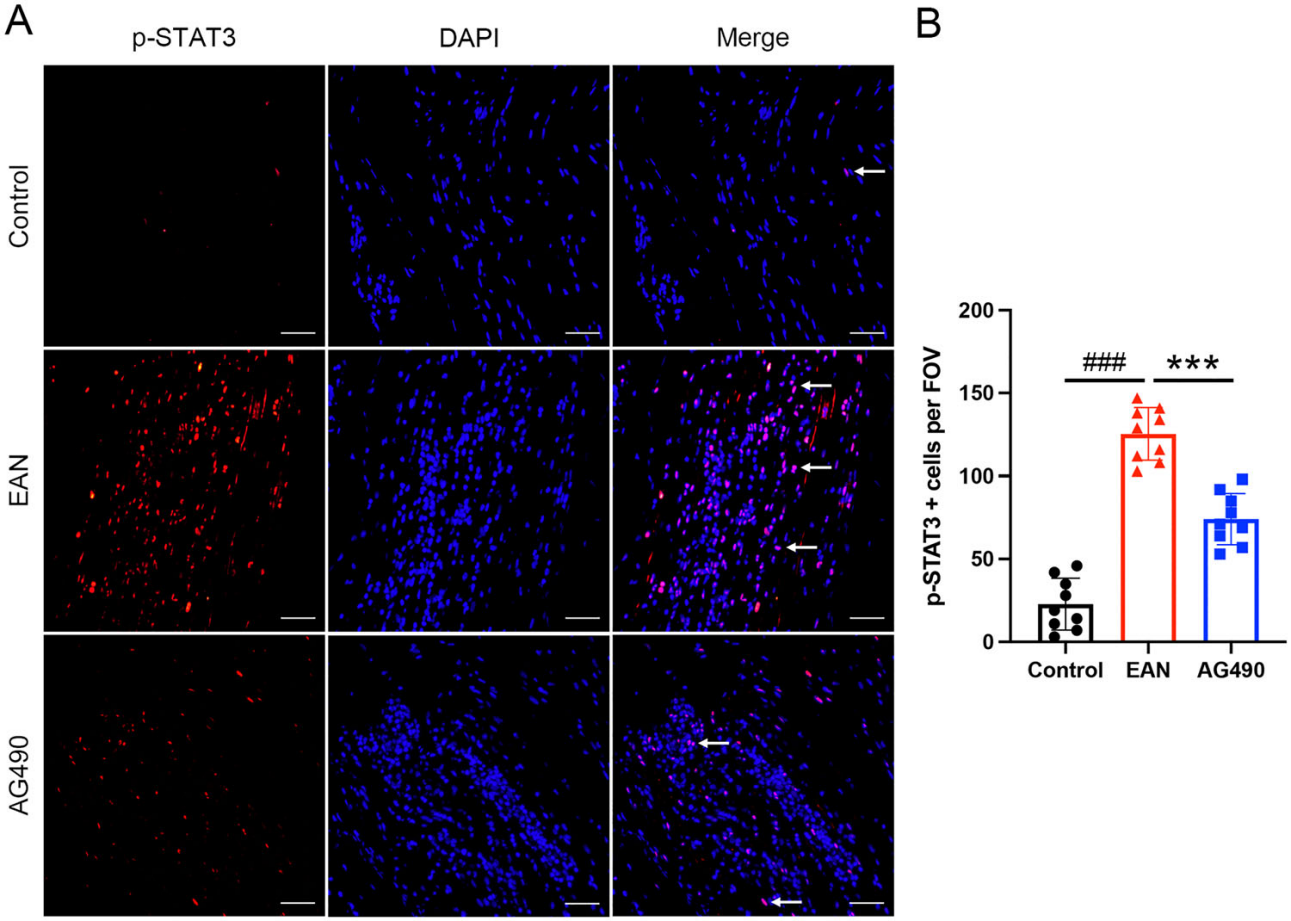
Effect of AG490 on the expression of p‐STAT3 in the sciatic nerves. (A) Representational photomicrographs of p‐STAT3 in the sciatic nerve of each group. p‐STAT3 positive cells were indicated by arrows. (B) The quantitative analysis of p‐STAT3 positive cells per FOV. FOV, field of view. Scale bars: 50 µm. Mean ± SD, *n* = 9/group. **###** *p *< 0.001, Control versus EAN. *** *p *< 0.001, AG490 versus EAN.

To further investigate the role of the JAK2‐STAT3 signaling pathway in EAN, the expression of related proteins was assessed by western blotting. The expression of JAK2, p‐JAK2, and p‐STAT3 was significantly upregulated in EAN rats. However, total JAK2 expression and the phosphorylation of JAK2 and STAT3 were decreased in rats treated with AG490 (Figure 3C–E). Furthermore, immunofluorescence showed high expression of p‐JAK2 and p‐STAT3 in EAN rats; however, after the treatment with AG490, a significant decrease in the expression of p‐JAK2 and p‐STAT3 in the sciatic nerve was observed (Figure 4A,B and Figure 5A,B). In conclusion, our findings suggested that inhibition of the JAK2/STAT3 signaling pathway could attenuate EAN in Lewis rats.

## 4 Discussion

This study aimed to investigate the function of the JAK2/STAT3 pathway and determine the therapeutic effect of AG490 in EAN rats. The results showed that AG490 treatment in EAN rats reduced the levels of the inflammatory cytokines IFN‐γ, IL‐6, and TGF‐β1 in the sciatic nerves, inhibited the activation of the JAK2‐STAT3 signaling pathway, reduced the inflammation and demyelination of the sciatic nerves, and resulted in functional improvement in EAN rats.

Cytokines play an important role in the occurrence and development of EAN and can be divided into pro‐ and anti‐inflammatory groups (Zhu et al. 1998; Hohnoki et al. 1998; Lu and Zhu 2011). Activated T cells and macrophages produce cytokines and proinflammatory cytokines such as tumor necrosis factor (TNF)‐α, IL‐6, and IL‐17 during the early phase of the disease, and the expression of anti‐inflammatory cytokines such as IL‐4 is upregulated during recovery stage of the disease (Lu and Zhu 2011).

IFN‐γ is secreted by Th1 cells and is the major marker of Th1 cells. IFN‐γ activates macrophages, promotes T‐cell differentiation to the Th1 type, and enhances the functions of other cytokines (Girkontaite et al. 2007; Schmidt et al. 1992). In GBS patients, the level of IFN‐γ in the serum is increased, and in an EAN model, the level of IFN‐γ is associated with the clinical course (Liu et al. 2020; Elkarim et al. 1998). The use of recombinant IFN‐γ could aggravate the clinical symptoms of EAN mice, and treatment with anti‐IFN‐γ antibodies improved clinical outcomes in GBS and EAN models. However, IFN‐γ knockout (KO) in mice could aggravate clinical symptoms by increasing the abundance of Th17 cells (Zhu et al. 2001). Whether IFN‐γ can serve as a target for GBS treatment needs further investigation.

TGF‐β1 is produced by Treg cells, antigen‐presenting cells (APCs), and B cells in vivo (Li et al. 2006). TGF‐β1 was shown to have therapeutic potential in systemic lupus erythematosus (SLE) because it induced the production of Tregs (Lu et al. 2008). TGF‐β1 blockade can prevent Th17 cell differentiation and the onset of EAE (Veldhoen et al. 2006). In the early stage of GBS, TGF‐β1 was expressed at a low level, partially due to immunomodulatory dysfunction in the early stages of the disease (Créange et al. 1998). A study performed in 2016 showed that the level of TGF‐β1 in the serum increased in GBS patients and was related to disease severity (Chang et al. 2016). An increase in the level of TGF‐β1 also occurred at the beginning of the recovery stage (Ossege et al. 2000).

IL‐6 is a proinflammatory cytokine that participates in multiple autoimmune diseases (Kallen 2002). The level of IL‐6 in the CSF and serum increased in GBS patients, and the same pattern was observed in EAN models (Oukka 2007; Sivieri et al. 1997; Zhu et al. 1994). The injection of recombinant IL‐6 into the sciatic nerve of adult Lewis rats worsened inflammation and demyelination, which indicated that IL‐6 may be an important factor at the beginning of the disease (Deretzi et al. 1999).

The JAK‐STAT signaling pathway is a classical signaling pathway involved in cell communication and body growth, and multiple cytokines act through the JAK‐STAT pathway (Philips et al. 2022). Cytokines bind to their receptors and activate JAK kinases, leading to their phosphorylation and dimerization, and phosphorylated JAK activates STAT to induce its phosphorylation. Phosphorylated STAT enters the nucleus and regulates the expression of genes (Bastian et al. 2019; Huang et al. 2022; O'Shea et al. 2015). The JAK‐STAT signaling pathway is involved in many autoimmune diseases, such as rheumatoid arthritis, ulcerative colitis, and SLE (Banerjee et al. 2017). The JAK inhibitors tofacitinib and ruxolitinib are approved for clinical practice.

The IL‐6/JAK2/STAT3 pathway participates in the occurrence of multiple diseases, and its main effect is achieved by modulating T‐cell differentiation. IL‐6 activates its downstream signaling pathway by activating JAK2 kinase (Philips et al. 2022). The IL‐6/JAK2/STAT3 pathway was activated in EAN rats, suggesting that the IL‐6/JAK2/STAT3 pathway participated in the development of the disease. The levels of IL‐6, p‐JAK2, and p‐STAT3 were lower in rats after treatment with AG490, indicating that inhibition of the IL‐6/JAK2/STAT3 pathway could weaken the inflammatory response of EAN rats. The levels of JAK2 and STAT3 were also increased in EAN rats, and surprisingly, the level of JAK2 decreased after AG490 treatment, but the STAT3 level showed no significant difference between EAN and AG490‐treated rats. This finding suggested that AG490 not only inhibited the phosphorylation of JAK2, but also inhibited the expression of total JAK2.

Our findings demonstrated that the IL‐6/JAK2/STAT3 pathway plays a role in the development of the EAN model induced by P2 peptide in male Lewis rats. Treatment with AG490 reduced inflammatory responses and alleviated disease severity at the nadir of EAN by inhibiting the IL‐6/JAK2/STAT3 pathway. The study indicated that the JAK2/STAT3 signaling pathway may be involved in the pathogenesis of the demyelinating subtype of GBS, and AG490 treatment might potentially benefit GBS patients.

There are some limitations in our research due to the experimental conditions. For example, a lack of electrophysiologic analysis and a rat cytokine protein array. Experimental methods should be improved in future research, such as preparing semi‐thin plastic‐embedded sections stained with toluidine blue to examine peripheral nerve morphology. Moreover, administering AG490 during the effector phase when rats exhibit symptoms, and conducting in‐depth investigation of the therapeutic mechanisms of AG490 in EAN, are crucial for translating the efficacy of AG490 from EAN models to potential therapeutic applications in GBS patients. In subsequent research, we will intervene in the expression of IL‐6 and investigate the downstream effects of STAT3 in EAN. In addition, further studies involving GBS patients are necessary to determine whether AG490 could be clinically beneficial for this patient population.

## Author Contributions

**Rumeng Zhou**: conceptualization, data curation, investigation, formal analysis, validation, writing–original draft. **Shuping Liu**: conceptualization, funding acquisition. **Yue Liu**: software, methodology. **Yin Liu**: methodology, software. **Rong Fu**: methodology, software. **Jiajia Yao**: conceptualization, investigation, supervision, data curation, writing–review and editing. **Zuneng Lu**: project administration, resources, supervision, writing–review and editing.

## Ethics Statement

All experimental protocols of this study were approved by the ethics committee of Renmin Hospital of Wuhan University. All methods are reported in accordance with ARRIVE guidelines (https://arriveguidelines.org) for the reporting of animal experiments.

## Consent

All authors have read and approved the final version of the manuscript and agreed to submit it for publication.

## Conflicts of Interest

The authors declare no conflicts of interest.

### Peer Review

The peer review history for this article is available at https://publons.com/publon/10.1002/brb3.70566

## Data Availability

The data that support the findings of this study are available on request from the corresponding author, upon reasonable request.
